# Commentary: Epigenetic Regulation of Phosphodiesterases 2A and 3A Underlies Compromised β-Adrenergic Signaling in an iPSC Model of Dilated Cardiomyopathy

**DOI:** 10.3389/fphys.2016.00418

**Published:** 2016-09-23

**Authors:** Lauren A. Cole, Jonathan H. Dennis, P. Bryant Chase

**Affiliations:** Department of Biological Science, Florida State UniversityTallahassee, FL, USA

**Keywords:** heart, dilated cardiomyopathy, troponin T, induced pluripotent stem cell, nucleus, histone methylation, epigenetic gene regulation

Wu et al. (2015) describe pioneering work that utilizes patient-derived induced pluripotent stem cells (iPSCs) from dilated cardiomyopathy (DCM) patients (Sun et al., 2012) and matched non-DCM relatives to study cellular mechanisms of DCM pathogenesis. They find that iPSC cardiomyocytes have proper β-adrenergic signaling while iPSCs from DCM patients exhibit impaired response to β-adrenergic agonist isoproterenol (ISO), which, physiologically, would be expected to compound the mechanical deficit associated with a mutation in troponin T (TnT). Surprisingly, Wu et al. (2015) find that the mechanisms of altered β-adrenergic signaling involve a direct role for TnT in epigenetic control of phosphodiesterase (PDE) expression, and that the mutation affects TnT function not only in the myofilament lattice, but also in the nucleus. This foundational work demonstrates the utility of iPSC-CMs for direct comparison of healthy vs. diseased tissues by providing a platform for identifying previously unrecognized molecular and cellular mechanisms in the progression of DCM.

The mutation studied by Wu et al. (2015) is a point mutation in the gene for the cardiac isoform of TnT, resulting in a single amino acid change (TNNT2 R173W) in or adjacent to TnT's tropomyosin-binding region. Many DCM mutations in myofilament proteins affect muscle function by decreasing Ca^2+^-sensitivity (e.g., when assaying Ca^2+^-dependent myofibrillar MgATPase activity, sliding speed of reconstituted thin filaments in motility assays, or force generation by permeabilized muscle preparations; Willott et al., 2010; Watkins et al., 2011); in other words, more cytoplasmic Ca^2+^ would be required to achieve the same functional response. This is indeed the case for the TNNT2 R173W mutation which shifts Ca^2+^sensitivity of myosin S1 MgATPase activity rightward (toward higher [Ca^2+^]) by almost 0.1 pCa units, with little or no effect on the maximum MgATPase activity or the maximum sliding speed of thin filaments in motility assays (Sommese et al., 2013). This altered Ca^2+^-responsiveness of the myofilaments almost certainly results directly in reduced mechanical function of the heart during systole, to the detriment of the DCM patient. Remodeling of the DCM heart, however, depends in part on changes in gene expression. Mechanisms of altered gene regulation in cardiomyopathies have typically focused on changes in Ca^2+^-signaling, mechanosensing, and/or energy metabolism (Frey et al., 2004; Ahmad et al., 2005; Kataoka et al., 2007; Lakdawala et al., 2012; Moore et al., 2012; LeWinter and Granzier, 2014). Wu et al. (2015) invoke a novel and more direct role of TnT in gene regulation.

Wu et al. (2015) found that TnT was present in one-third of nuclei from iPSCs derived from DCM patients with the TNNT2 R173W mutation, compared to ~5% of nuclei of iPSCs derived from normal individuals. TnT is an abundant myofilament protein present in the sarcomere, responsible for attachment of the troponin complex to tropomyosin and transmission of the Ca^2+^ signal that activates systolic cardiac contraction (Figure 1). Although TnT contains a strong nuclear localization signal (NLS), its functional role in the nucleus of striated muscle myocytes is poorly understood (Bergmann et al., 2009; Zhang et al., 2015, 2016). Identification of TnT interacting proteins in the nucleus is critical to understanding its function.

**Figure 1:**
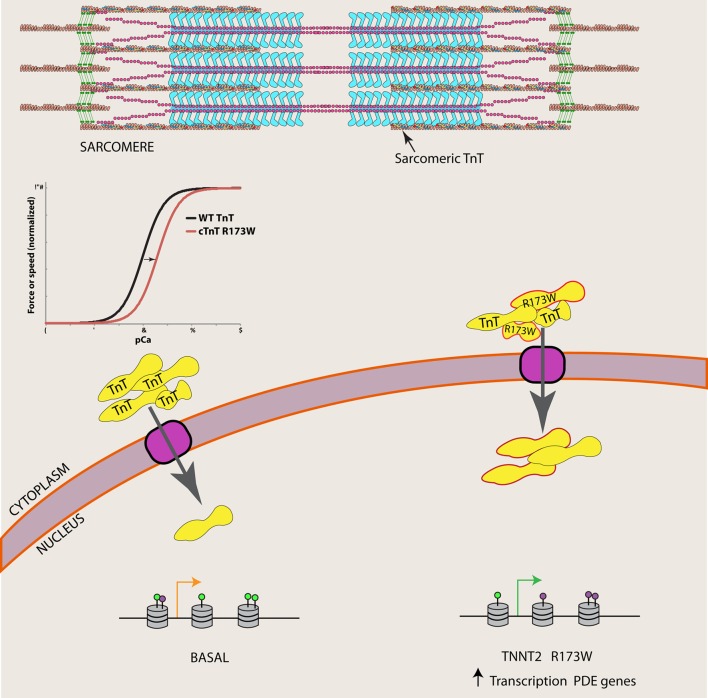
**The R173W mutation is associated with increased nuclear TnT in DCM patients**. Wu et al. (2015) show nuclear TnT is associated with demethylases, and catalog an altered epigenetic landscape of phosphodiesterase (PDE) genes in DCM iPSCs (purple lollipops represent H3K4me3 and green lollipops represent H3K27me3), which may lead to increased transcription of PDE genes in DCM patients.

Wu et al. (2015) performed co-immunoprecipitation studies in cardiomyocyte nuclear extracts to identify TnT interacting proteins. They found that TnT is associated with histone demethylases KDM1A and KDM5A, as well as histone H3. Furthermore, they characterized chromatin patterns of the PDE 2A and 3A genes, where the authors found significant increases of activation marks (H3K4me3) and decreased repressive marks (H3K27me3) in sequences defined by the authors as regions 1 and 2. Assuming high specificity for the various antibodies used throughout their assays, these results suggest that TnT normally plays a role in the epigenetic regulation of at least these PDE genes. Their study furthermore demonstrates that a TnT mutation not only affects sarcomeric function, but also contributes to the improper regulation of both nuclear localization of TnT and PDE gene expression in DCM patients (Figure 1). Precise epigenetic regulation of cardiomyocyte differentiation as well as regulation of expression in a cell-type-specific manner has been recently documented, demonstrating this layer of information is critical for understanding cardiomyocyte (dys)function (Paige et al., 2012; Wamstad et al., 2012; O'Meara and Lee, 2015; Preissl et al., 2015). An improper epigenetic landscape likely contributes to inappropriate regulation of many genes, and it will be important for future work to explore other known DCM mutations in the context of genome architecture.

Wu et al. (2015) demonstrate the use of iPSCs to study a prevalent heart disease and determine a novel role of epigenetic regulation in pathogenesis of DCM. This finding demonstrates that mutations in mechanical proteins that lead to DCM pathogenesis via sarcomere dysfunction can also be exacerbated by regulation of epigenomic state. Nuclear localization of cardiac troponin I (TnI), cardiac troponin C (TnC), and cardiac TnT has been shown in rat neonatal ventricular cardiomyocytes, but their relationship with one another, and presumably tropomyosin and actin, in the nucleus has yet to be clearly established (Asumda and Chase, 2012). Interestingly, co-IP data from Wu et al. (2015) did not identify TnI or tropomyosin as interacting partners of nuclear TnT. It may be the case that these partners in thin filament regulation have independent roles in the nucleus. Because these proteins are often mutated in DCM patients, further studies are necessary to not only delineate the function of these proteins in the nucleus in normal individuals, but to determine whether the unique mechanisms identified by Wu et al. (2015) (i.e., unexpected changes in nuclear localization and unexpected interactions with other molecules, which in this instance affect epigenetic regulation of physiologically important genes) are specific only to the R173W mutation in TNNT2, or if they are more commonly associated with other myofilament protein mutations and other mutations that cause cardiomyopathies (Schoffstall et al., 2011; Chase et al., 2013; Hershberger et al., 2013; Teekakirikul et al., 2013; Brunet et al., 2014; Ho et al., 2015; Teo et al., 2015). The involvement of nuclear mechanical proteins in regulation of chromatin, and thus expression, is a new and important aspect of DCM pathogenesis.

## Author contributions

LC and PC wrote and edited the commentary, JD edited the commentary.

### Conflict of interest statement

The authors declare that the commentary was written in the absence of any commercial or financial relationships that could be construed as a potential conflict of interest. The reviewer BB and handling Editor declared their shared affiliation, and the handling Editor states that the process nevertheless met the standards of a fair and objective review.
